# Creation of an Open-Access Lung POCUS Image Database for Deep Learning and Neural Network Applications

**DOI:** 10.24908/pocusj.v11i01.19439

**Published:** 2026-04-22

**Authors:** Andre Kumar, Pawan Nandakishore, Alexandra June Gordon, Evan Baum, Jai Madhok, Youyou Duanmu, John Kugler

**Affiliations:** 1 Department of Medicine, Stanford University School of Medicine, Stanford, CA, USA; 2 Department of Emergency Medicine, Stanford University School of Medicine, Stanford, CA, USA; 3 Department of Anesthesiology, Stanford University School of Medicine, Stanford, CA, USA

**Keywords:** Point of care ultrasound, Lung POCUS, Lung Ultrasound, Diagnosis, Pneumonia, Artificial Intelligence, Deep Learning, Machine Learning

## Abstract

**Background:**

Lung point of care ultrasound (POCUS) offers advantages over traditional imaging for diagnosing pulmonary conditions, with superior accuracy compared to chest X-ray and lower cost compared to computed tomography. Despite these benefits, widespread adoption is limited by operator dependency, moderate interrater reliability, and training requirements. Deep learning (DL) could potentially address these challenges, but the development of effective algorithms is hindered by the scarcity of comprehensive image repositories with proper metadata.

**Methods:**

We created an open-source dataset of lung POCUS images derived from a multi-center study involving 226 adult patients presenting to emergency departments with respiratory symptoms between March 2020 and April 2022. Images were acquired using a standardized scanning protocol (12-zone or modified 8-zone) with various POCUS devices. Three blinded researchers independently analyzed each image following consensus guidelines, with disagreements adjudicated to provide definitive interpretations. Videos were preprocessed to remove identifiers, and frames were extracted and standardized to 512×512 pixels using letterboxing to maintain aspect ratios.

**Results:**

The dataset contained 1,871 video clips comprising 324,027 frames extracted and standardized to 512×512 pixels. Half of the participants (50%) had COVID-19 pneumonia. Among all clips, 66% contained no abnormalities, 18% contained B-lines, 4.5% contained consolidations, 6.4% contained both B-lines and consolidations, and 5.2% had indeterminate findings. Pathological findings varied significantly by lung zone, with anterior zones more frequently normal and less likely to show consolidations compared to lateral and posterior zones.

**Discussion:**

This dataset represents a large, annotated lung POCUS repository and includes patients with and without COVID-19. The repository metadata and expert interpretations enhance its utility for DL applications. Despite limitations including potential device-specific characteristics and COVID-19 predominance, this repository provides a valuable resource for developing artificial intelligence tools to improve lung POCUS acquisition and interpretation.

## Introduction

Lung point of care ultrasound (POCUS) offers significant advantages as a bedside tool to diagnose several pulmonary conditions, including pneumonia, pulmonary edema, pleural effusion, pneumothorax, and interstitial disease. It can be expediently performed at the point of care to enhance diagnostic accuracy and monitor disease progression [1,2]. Lung POCUS has demonstrated superior diagnostic accuracy compared to chest X-ray for identifying pneumonia, pulmonary edema, pleural effusion, and pneumothorax [1,3–5]. When compared to computed tomography (CT), lung POCUS often demonstrates similar diagnostic performance for these conditions [6–8]. Lung POCUS devices are more cost-effective than traditional imaging equipment such as X-ray or CT machines, making them particularly valuable in resource-limited settings or overwhelmed clinical environments. Despite these advantages, widespread lung POCUS adoption faces several challenges, including operator dependency for image acquisition, moderate interrater reliability for interpreting pathological findings, and the need for standardized provider training [9,10].

Deep learning (DL), a subfield of artificial intelligence (AI), has the potential to address many obstacles hindering lung POCUS adoption [11]. DL utilizes machine learning to automatically characterize features from raw data [11–13]. By analyzing large repositories of POCUS images, DL can make predictions about new images [13]. Potential applications of DL in POCUS include aiding examiners in image acquisition, providing anatomical labelling of structures, assessing image quality, identifying pathological findings, and assisting with interpretation [11–14]. Through these capabilities, DL may improve lung POCUS image quality, automate analysis, and help healthcare professionals make more accurate and timely clinical decisions [11–13].

Despite the potential benefits of combining lung POCUS with DL applications, there is a scarcity of high-quality image repositories needed to develop effective DL algorithms [15–17]. There is a critical need to create open-source image libraries that can be used for current and future applications of POCUS. Many existing databases derive primarily from inpatients, and relatively few datasets contain healthy controls [15,16]. Furthermore, these repositories often lack important metadata such as patient characteristics, scan locations, expert interpretations, and assessments of image quality [15].

In this manuscript, we describe the creation of an open-source dataset of lung POCUS images derived from 226 patients, comprising 303,977 individual video frames. This dataset is one of the largest lung POCUS repositories to date and includes metadata regarding patient information, expert interpretations of findings, and control cases. Our dataset includes additional metadata to further aid in applications of DL as applied toward lung POCUS.

## Methods

### Dataset Derivation

All patient images were collected as part of a multi-center prospective cohort study between 3/2020 and 4/2022 (Clinicaltrials.gov Registration: NCT04384055) [18]. Inclusion criteria were adults presenting to the emergency department with respiratory symptoms concerning for COVID-19. For healthy controls, patients presenting to the emergency department or clinic without known pulmonary disease or symptoms were included. Patients were excluded if they did not receive a lung POCUS examination. Patient demographics and clinical information were collected. This study received institutional review board approval, including for the creation of the de-identified image database (Protocol 74680).

### Scanning Protocol

All images were acquired utilizing a previously-published standardized scanning protocol [18]. Physicians were instructed to use a 12-zone scanning protocol for lung POCUS views. If a 12-zone protocol could not be performed due to the patient's condition (inability to turn, patient discomfort), then a modified 8-zone protocol capturing the anterior and lateral lung fields was performed. The probe marker was oriented cranially. The 12-zone lung POCUS protocol involved obtaining two anterior, two lateral, and two posterior views obtained on each hemithorax. The 8-zone protocol involved two anterior and two lateral zones on each hemithorax. The two views for each location (e.g., the two anterior views) were obtained as cranial and caudal views, with the nipple line as a bisecting line to distinguish between the two areas. Scans were obtained in supine or semi-recumbent position.

This study utilized several cart-based and handheld POCUS devices, including Butterfly IQ^TM^ (Burlington, MA), Vave^TM^ (Santa Clara, CA), Fujifilm Sonosite LX^TM^ (Bothell, WA), GE Venue^TM^ (Chicago, IL) and Echonous^TM^ (Redmond, WA), which represent the commercially available POCUS devices at our institutions. All devices used a phased array probe and were set to the “lung” preset. The lung preset varied between machines, but in general, this preset provided optimal depth, gain, and frame rate to visualize lung artifacts (B-lines, consolidations, and effusions). A total of 226 participants were enrolled in the study.

### Interpretation

Physician interpretation of the images was performed using a previously developed consensus guideline for lung POCUS images [19]. Briefly, B-lines were defined as vertically oriented artifacts originating from the pleura that extend at least 12 cm and erase A-lines as they move over them. Subpleural consolidations included irregular or thickened pleura and hyper echogenicity inferior to the pleura. Lobar consolidations were defined as dense, “hepaticized” lung. Physicians who interpreted the images met the following criteria: 1) at least five years of faculty experience, 2) previously credentialed in POCUS at our institution, and 3) completed a POCUS certificate program or advanced fellowship in POCUS. Three researchers (AK, JK, YD), who were blinded to any patient information, independently analyzed each image and provided their interpretation on separate electronic spreadsheets (sample provided in Supplementary Material S1). When disagreements occurred, the researchers met to discuss their interpretations and the underlying imaging features to reach consensus through dialogue. If no consensus could be reached, the interpretation for that image was marked as “indeterminate.” Previous investigations have demonstrated moderate to substantial interrater reliability for lung POCUS across different experience levels and probe types [20,21]. Within this dataset, there was initial agreement in 77% of the clips (N = 1,445), with 23% (N = 426) requiring adjudication, which is consistent with our previous findings where our interpretation protocol had moderate-to-substantial interrater reliability for the lung POCUS findings included in this study (k = 0.79 for normal scans, k = 0.79 for B-lines, k = 0.57 for consolidations) [10].

### Image Preprocessing

**Figure 1.**
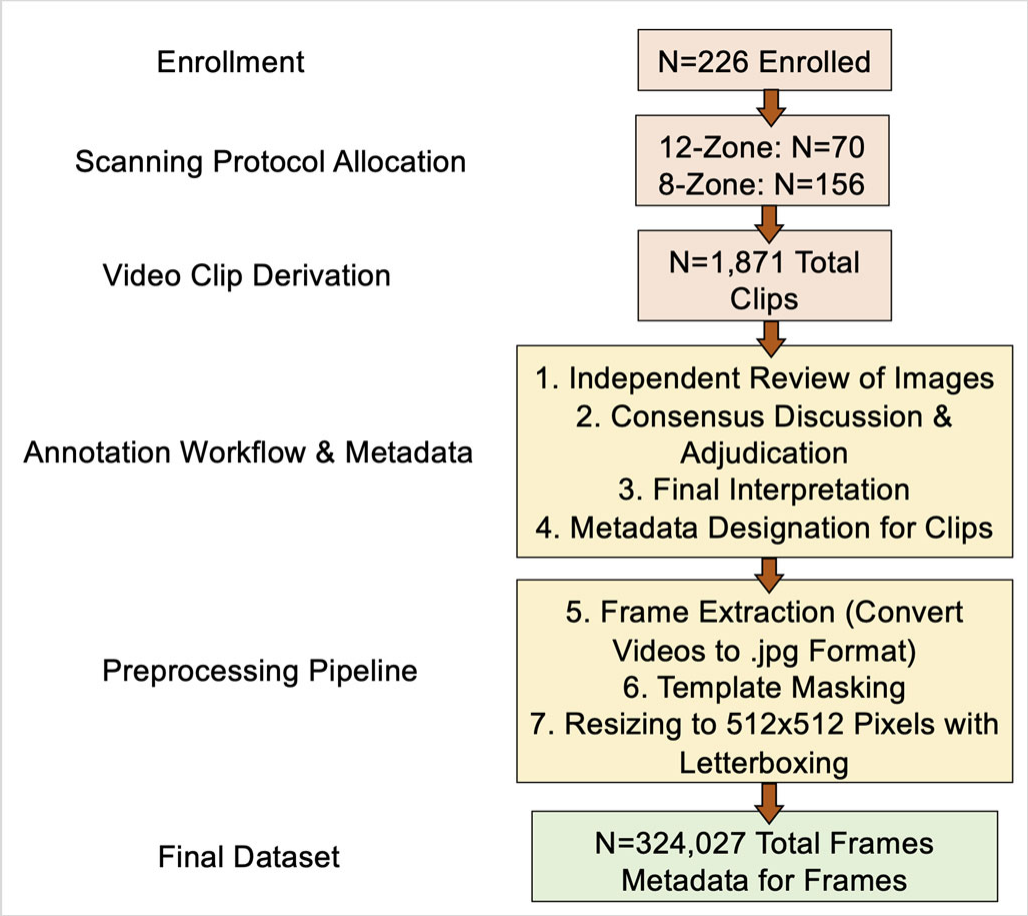
Study Enrollment, Annotation, and Preprocessing Pipeline.

All 1,871 video clips underwent systematic preprocessing to create a standardized dataset suitable for DL applications (Figure 1). Videos were first reviewed to identify and remove patient identifiers. Using OpenCV (version 3.12.0) [22], we extracted individual frames from each video at the native frame rate. Template masking was applied to remove device-specific annotations and identifiers while preserving the ultrasound image field. Frames were then standardized to 512×512 pixels using letterboxing to maintain aspect ratios, resulting in 324,027 total frames (Figure 1). Each frame was saved in .jpg format with a standardized nomenclature indicating participant identification, lung zone, and frame number (e.g., P001_Zone1_Frame005.jpg). This preprocessing created a dataset suitable for convolutional neural network architectures, for image classification tasks.

### Variable Definitions

All patient diagnoses were determined through retrospective chart reviews of the primary treating physician's discharge summary or emergency department note. COVID-19 infection testing was obtained via nasopharyngeal polymerase chain reaction (PCR) in all patients presenting to the emergency department.

## Results

The open access lung POCUS repository and associated metadata can be found at: https://github.com/kumarandre/OpenPOCUS.

The dataset consisted of 1,871 video clips (324,027 frames) derived from 226 patients (Table 1). Of these participants, 114 (50%) were diagnosed with COVID-19 pneumonia at the time of their scan. Additional diagnoses are presented in Table 1. The mean body mass index (BMI) was 28.4 (SD 6.8), and 90 patients (40%) were female.

**Table 1.** Patient Demographics and Scan Characteristics. Other diagnoses included diarrhea N = 2, osteomyelitis N = 2, delirium N = 2, failure to thrive N = 3, stroke N = 1, overdose N = 1, acute coronary syndrome N = 1, chest trauma N = 4, and pleural effusion N = 2.

**Patients, N**	226
**Video Clips, N**	1874
**Devices Used**	
Butterfly, N (%)	134 (60)
Echonous, N (%)	21 (9.3)
Sonosite, N (%)	1 (0.4)
Vave, N (%)	56 (25)
GE Venue, N (%)	14 (6.2)
**BMI, Mean (SD)**	28.4 (6.8)
**Median Age (IQR)**	54 (35–70)
Age 18–30, N (%)	6 (3.0)
Age 31–50, N (%)	83 (42)
Age 51–70, N (%)	67 (34)
Age 71–90, N (%)	39 (20)
Age >90, N (%)	5 (3.0)
**Female, N (%)**	90 (40)
**Diagnosis**	
COVID, N (%)	114 (50)
Bacterial Pneumonia, N (%)	12 (5.3)
Viral Pneumonia (non-COVID), N (%)	12 (5.3)
Obstructive Airway Disease, N (%)	21 (9.3)
Healthy Control, N (%)	36 (16)
Pulmonary Edema, N (%)	14 (6.2)
Other, N (%)	17 (7.5)

Regarding scanning protocols, 70 patients (31%) had scans obtained using the 12-zone protocol, while 156 patients (69%) had scans using the 8-zone protocol. Among all scans, 1,233 clips (66%) contained no abnormal findings, 338 clips (18%) contained any count of B-lines, 84 clips (4.5%) contained consolidations, 119 clips (6.4%) contained both B-lines and consolidations, and 97 clips (5.2%) had indeterminate findings (Supplementary Material S1).

The dataset included additional metadata such as COVID-19 status, normal versus abnormal interpretation, and expert interpretation of each video clip for a given lung zone (Table 2).

**Table 2.** Scan Counts and Findings. Each lung zone is displayed with the number of scans and findings.

	Total	Zone 1	Zone 2	Zone 3	Zone 4	Zone 5	Zone 6	Zone 7	Zone 8	Zone 9	Zone 10	Zone 11	Zone 12
Scans	1871	219	198	191	197	70	70	219	195	186	189	69	68
Normal	1233	154	137	123	136	43	39	144	140	106	122	46	43
B-lines	338	42	35	31	24	15	12	50	30	40	31	17	11
Consolidation	84	4	9	12	14	4	10	5	0	10	7	2	7
B-lines & Consolidation	119	17	15	19	6	3	4	14	5	19	11	1	5
Indeterminate	97	2	2	6	17	5	5	6	20	11	18	3	2

## Discussion

We describe the creation of a large lung POCUS dataset derived from adult patients with annotated metadata for each lung clip. This dataset encompasses patients with various pulmonary diseases as well as healthy controls. With over 320,000 individual frames, this open-source repository is among the largest available. The dataset can be used to aid in future applications of AI as applied toward lung POCUS and medical imaging. Specific applications include the development of neural network models to classify and segment lung POCUS images for pathology detection and the determination of normal versus abnormal. Additionally, it represents a dataset that can be used for individuals to test their current models to provide verification of their accuracy.

Previous investigations have found moderate to substantial interrater reliability for lung POCUS findings, which is an important consideration given the expert over-reads of this study [10,20,21]. Within our group, we have observed similar interrater reliability for the lung POCUS findings in this database [10]. For this project, we implemented an adjudication process to provide a single interpretation for future DL applications. Imaging findings that have poor interrater reliability may result in difficulty establishing a singular ground truth, as human labellers are needed to train these models. Therefore, models developed around findings with higher reliability (e.g., normal findings with κ = 0.79 or B-lines with κ = 0.79) are more likely to achieve greater accuracy than models targeting less reliable findings. We report on interrater reliability for our dataset to inform future applications of these images and highlight potential accuracy limitations. As DL and neural network integration become more prominent in POCUS, determining how these technological advances can aid providers in image acquisition and interpretation will be crucial [11,13]. It will be equally important to compare the accuracy of these models with expert physicians, given that interpretations among providers vary [10,21,23].

Although other open-source lung POCUS databases have been described, our dataset represents the largest to date [24]. Our dataset offers unique advantages over other existing open-source lung POCUS databases [15–17,23]. For example, the COVIDx-US dataset (v1.5) contains 242 videos and more than 29,000 preprocessed images with video-level annotations. Our dataset provides a larger number of source videos (1,871 clips) with frame-level preprocessing (324,027 frames at 512×512 pixels), comprehensive metadata including COVID-19 status, and most importantly, a balanced mix of COVID-19 (50%) and non-COVID-19 pathologies. While COVID-19-predominant datasets face declining clinical relevance as the pandemic evolves, the heterogeneous manifestations of SARS-CoV-2 on lung parenchyma (including B-lines, consolidations, pleural irregularities, and mixed patterns) make it an ideal teaching model for lung POCUS interpretation. The pathophysiologic findings of COVID-19 pneumonia overlap substantially with other acute respiratory conditions, providing a rich substrate for training computer vision models to recognize fundamental lung POCUS artifacts. Furthermore, approximately half of our participants had non-COVID-19 conditions, providing broader generalizability than purely COVID-focused datasets [15–17,23].

It is important to note that there may be subtle differences in lung POCUS findings between COVID-19 and other conditions. Models trained primarily on COVID-19 may miss these distinctions, particularly if used for diagnostic purposes [12,23,24]. For example, cardiogenic pulmonary edema (due to heart failure) and non-cardiogenic pulmonary edema (related to acute respiratory distress syndrome) present differently on lung POCUS [25]. Since COVID-19 typically causes imaging findings consistent with non-cardiogenic pulmonary edema, overreliance on COVID-19-derived models may reduce reliability in future imaging applications [25].

## Limitations

This study has several limitations. We utilized only three reviewers for the interpretation of each video clip, which may reduce the accuracy of findings. Analysis was performed at the clip level rather than the frame level, requiring caution when extrapolating metadata to individual frames. Approximately half of the scans were derived from patients with COVID-19, which potentially limits generalizability to specific disease models. The majority of scans (59%) were obtained using a single device (Butterfly IQ™) in a phased array setting without the use of a linear probe, which has additional diagnostic benefits in lung POCUS. Despite preprocessing to remove device-specific markings, each ultrasound probe has unique characteristics (scan angle, frame rate, gray-scaling), potentially limiting model generalizability across devices. The predominance of scans from a single device (59% Butterfly IQ™) may introduce device-specific characteristics despite our preprocessing efforts to remove identifying markers. Future work should validate models across diverse ultrasound platforms.

## Conclusions

We present a large, open-source lung POCUS database derived from diverse patients, including healthy controls. The methods used in creating this dataset can serve as a template for future datasets. Importantly, imaging datasets should be built on well-defined patient populations, which in this study included adults presenting to the emergency department with respiratory symptoms. Images should be reviewed by experts to label findings, and diagnoses should be assigned whenever possible. With the addition of similar lung POCUS databases, it is possible to develop image interpretation tools powered by DL to assist in the acquisition and interpretation of lung POCUS.
