# Supplementary material for: Creation of an Open-Access Lung POCUS Image Database for Deep Learning and Neural Network Applications
Source: POCUS J. 2026 Apr 22;11(1):62–7. doi: 10.24908/pocusj.v11i01.19439 (PMC13161784; doi:10.24908/pocusj.v11i01.19439)
Supplement: Supplementary file 1 [file pocusj-11-01-19439-s001.pdf]

Supplementary Material S1. Pathology by Lung Areas.

	Pathology (N)				
Lung Zone	Normal	B-Lines	Consolidation	Both	Indeterminate
Anterior	575	157	18	51	30
Lateral	505	126	43	55	52
Posterior	171	55	23	13	15
Right	601	179	31	55	60
Left	632	159	53	64	37
